# Genetic dissection of developmental responses of agro-morphological traits under different doses of nutrient fertilizers using high-density SNP markers

**DOI:** 10.1371/journal.pone.0220066

**Published:** 2019-07-23

**Authors:** Anumalla Mahender, Jauhar Ali, G. D. Prahalada, Ma. Anna Lynn Sevilla, C. H. Balachiranjeevi, Jamaloddin Md, Umer Maqsood, Zhikang Li

**Affiliations:** 1 Rice Breeding Platform, International Rice Research Institute, Los Baños, Manila, Philippines; 2 Strategic Innovation Platform, International Rice Research Institute, Los Baños, Manila, Philippines; 3 Agricultural Biotechnology Division, National Institute for Biotechnology and Genetic Engineering, Pakistan; 4 Chinese Academy of Agricultural Sciences, Haidian District, P.R. China; Institute of Genetics and Developmental Biology Chinese Academy of Sciences, CHINA

## Abstract

The production and productivity of rice (*Oryza sativa* L.) are primarily influenced by the application of the critical nutrients nitrogen (N), phosphorus (P), and potassium (K). However, excessive application of these fertilizers is detrimental to the environment and increases the cost of production. Hence, there is a need to develop varieties that simultaneously increase yields under both optimal and suboptimal rates of fertilizer application by maximizing nutrient use efficiency (NuUE). To unravel the hidden genetic variation and understand the molecular and physiological mechanisms of NuUE, three different mapping populations (MPs; BC_1_F_5_) derived from three donors (Haoannong, Cheng-Hui 448, and Zhong 413) and recipient Weed Tolerant Rice 1 were developed. A total of three favorable agronomic traits (FATs) were considered as the measure of NuUE. Analysis of variance and descriptive statistics indicated the existence of genetic variation for NuUE and quantitative inheritance of FATs. The genotypic data from single-nucleotide polymorphism (SNP) markers from Tunable Genotyping-By-Sequencing (tGBS) and phenotypic values were used for locating the genomic regions conferring NuUE. A total of 19 quantitative trait loci (QTLs) were detected, out of which 11 QTLs were putative on eight chromosomes, which individually explained 17.02% to 34.85% of the phenotypic variation. Notably, *qLC-II_1* and *qLC-II_11* detected at zero fertilizer application showed higher performance for LC under zero percentage of NPK fertilizer. The remarkable findings of the present study are that the detected QTLs were associated in building tolerance to low/no nutrient application and six candidate genes on chromosomes 2 and 5 within these putative QTLs were found associated with low nutrient tolerance and related to several physiological and metabolic pathways involved in abiotic stress tolerance. The identified superior introgressed lines (ILs) and trait-associated genetic regions can be effectively used in marker-assisted selection (MAS) for NuUE breeding programs.

## Introduction

Ensuring global food security is a significant concern for the rapidly growing world population that is expected to reach 9.8 billion by 2050 [1]. This situation concerning food security is even gloomier in developing countries. To meet the global food demand mainly in Asia, there is a tremendous need to increase rice production by 60–70% by 2050 [2]. At present, the core point of attention for scientists and policymakers is to develop different strategies for improving average yield productivity for sustainability. Plant breeders and biotechnologists follow various strategies to develop varieties with biotic stress resistance, abiotic stress tolerance, and multi-nutrients against malnutrition and cultivars with higher nutrient use efficiency, with the ultimate goal of developing climate-smart rice varieties with higher grain production [3–6]. Several biotic and abiotic stresses, decreasing arable land, labor unavailability, high cost of input fertilizers, water scarcity, and deficiency of nutrient elements in the soil are the major limiting factors for the development of multiple-stress-tolerant, input-use-efficient, and climate-smart rice cultivars [7–11]. Since the beginning of the green revolution, the nutrient elements nitrogen (N), phosphorus (P), and potassium (K) are the key components that maintain nutritional status through an oxidation process and they are significantly associated with increasing global crop productivity [12,13]. These major nutrients play a vital role in cellular mechanisms, enzyme synthesis, and osmotic regulation, and represent several structural components for numerous metabolic pathways to maintain proper plant growth and development [14,15].

Judicious application of fertilizers is the key for sustainable crop production. The improper and unscientific application of fertilizers containing N, P, and K is not only a burden as an extra cost for farmers but is also associated with environmental and human hazards [9]. To date, extensive research in this area has confirmed that the application of higher input fertilizers can cause imbalances in nutrient status in the soil and hence damage soil fertility in a longer period [16,17]. Chinese farmers are applying a higher dose of N fertilizer, particularly in Jiangsu Province. A higher dose of fertilizer of about 305 kg ha^−1^ is being applied to obtain higher yield vis-à-vis the world’s average dose of N fertilizer at 180 kg ha^−1^ [18–20]. Recently, Feng et al. [21] mentioned that released rice varieties in China had a greater yield potential, up to 12 t ha^-1^ or even higher, under high input doses of fertilizer and water. However, a higher dose of fertilizer applications leads to the risk of increased pests and diseases, consequently increasing the cost of pesticide application [22]. To reduce the cost of production and address the environmental safety issues associated with excessive fertilizer application, an appropriate dosage is required to ensure higher yield. There are mainly two different management systems, agricultural and integrated nutrient management systems that determine nutrient uptake, and influences increase in the grain yield and minimize the environmental hazards [23–26]. Green Super Rice (GSR) at the International Rice Research Institute (IRRI) has greatly contributed to minimizing the excessive application of chemical fertilizers by developing rice varieties with high NuUE and that perform better with integrated nutrient management. To date, 42 GSR varieties have been released worldwide and are available for 11 countries in South Asia, Southeast Asia, and Southern and Eastern Africa. Furthermore, these GSR varieties not only showed stable higher yield under low input environments but also showed tolerance of multiple biotic and abiotic stresses [27–31]. More than 130 GSR breeding cultivars are currently undergoing national varietal testing (www.isaaa.org), out of which 60 are being tested in different All India Coordinated Rice Improvement Project (AICRIP) trials in India alone.

Systematic nutrient management studies, site-specific nutrient management, farmers’ fertilizer practices, real-time nitrogen management, fixed-time adjustable-dose nitrogen management, integrated soil-crop system management, and optimal nitrogen management have been tested to increase rice yield under low input conditions [19,20,32–35]. Importantly, a significant yield advantage of 1.51 t ha^-1^ was achieved by reducing the N fertilizer application by 22.41% [20]. Hence, an organized and convenient procedure is required to establish a stage-specific fertilizer dose application to improve the nutrient balance for increasing agronomic advantages with the least environmental hazards. Three key favorable agronomic traits (FATs), plant height (PH), tiller number (TN), and leaf chlorophyll content (LC), and other yield-attributing traits contribute significantly to better plant architecture [36–39]. These traits were highly influenced by the application of a combination of complete N, P, and K fertilizers. Interestingly, these traits are controlled by the combined effects of several genes (polygenes) with a large influence of environment. Hence, the dissection of these traits that follow non-Mendelian segregation had become difficult and complex, especially when applying traditional breeding tools and methodologies. Recent advances in molecular marker and genomics technology paved the way for dissecting the genetic basis of the complex inheritance of key agronomic traits. Genetic analysis of quantitative trait loci (QTLs) can estimate and identify the influences of different genes responsible for quantitative traits with high statistical power. Further, the identified genes conferring these quantitative traits can be introgressed through precise MAS either singly or in combination with other useful traits/genes through marker-assisted pyramiding approaches [21,40,41].

DNA-based molecular markers such as restriction fragment length polymorphism, amplified fragment length polymorphism, cleaved amplified polymorphic sequence, and simple sequence repeats were used to generate genotypic information to determine the genetic characteristics of the lines. However, the generation of information is time-consuming and labor-intensive [42–44] as these markers are slower in work to provide accurate genotypic information for predicting candidate gene loci due to the low resolution of QTL mapping, environment-dependent expression of QTLs, and gene epistasis [44,45]. Recently developed high-throughput sequencing technologies are robust, accurate, and more informative for generating millions of single nucleotide polymorphism (SNP) markers in a shorter period [43,46,47]. These SNPs are most promising to develop genetic linkage maps for QTL dissection of many traits with higher chromosomal coverage [48–51]. To date, several QTLs have been reported for N, P, and K deficiency tolerance traits by using different QTL mapping methods in different genetic backgrounds of MPs such as recombinant inbred lines (RILs), backcross inbred lines (BILs), introgression lines (ILs), doubled haploids (DHs), chromosome segment substitution lines (CSSLs), and BC_2_F_3_ families in rice [10,41,52]. According to a comprehensive literature survey and exploring information available in the Gramene database (http://archive.gramene.org), more than 150 QTLs for N, 130 QTLs for P, and 15 QTLs for K have been associated with deficiency tolerance traits in rice. These significant QTLs are located on eight chromosomes (1, 3, 4, 5 7, 8, 9, and 12) for N [21,53–59]. Similarly, P deficiency tolerance QTLs have been reported on five chromosomes (1, 2, 6, 11, and 12) [10,41,53,60–67]. K deficiency tolerance QTLs are located on three chromosomes (3, 5, and 8) [68,69]. Notably, rather than a deficiency of the individual nutrient, either N or P or K, the combination of the complete N, P, and K deficiency has a significant impact on agronomic traits such as tillering ability, PH, and LC, which are key contributors for determining total grain yield [70,71].

To the best of our knowledge, the identification of QTLs conferring individual N, P, and K deficiency tolerance has been less explored. Interestingly, there was no comprehensive information on QTL data regarding the effect of fertilizer application in different growth interval (DGI) stages and during complete deficiency of N, P, and K fertilizers in rice. Hence, understanding the genetic information of N, P, and K deficiency tolerance of FATs under DGI stages of fertilizer doses is crucial to finding suitable rice cultivars for N, P, and K use efficiency (NuUE). In the current study, we used a set of early backcross MPs derived from a cross of three donors, Haoannong (HAN), Cheng-Hui 448 (CH448), and Zhong 413 (Z413), with a recipient parent, Weed Tolerant Rice 1 (WTR-1), to study the genetic variation for deficiency tolerance for N, P, and K at different stages. In addition, we detected genomic regions conferring N, P, and K defficiency tolerance by using phenotypic evaluation under three different doses of N, P, and K fertilizer, 338.90 kg ha^-1^ (100%), 271.10 kg ha^-1^ (80%), and 0 kg ha^-1^ (0%), and genotyping by tGBS technology to detect the QTLs associated with the trait of low input tolerance. The major objectives of the study were to (i) identify the FAT responses under three different doses of fertilizer application, 100%, 80%, and 0% for N, P, and K conditions; (ii) understand the genetic basis of FAT responses at DGI stages under three different doses of fertilizer application through correlation and heritability estimation; (iii) identification of promising tolerant ILs; and (iv) detect the main-effect QTLs (M-QTLs) at DGI stages under different doses of fertilizer by also estimating the additive effects of M-QTLs (v) enumeration of the candidate genes within the major effect QTLs based on the in silico candidate genes analysis using online databases.

## Materials and methods

### Plant materials and developing mapping populations

The parental line WTR-1 developed by using a novel Green Super Rice breeding strategy, was used as the recurrent parent while HAN, CH448, and Z413 were used as donor parents [72–74] for the development of MPs for the identification of the genomic regions controlling N, P, and K use efficiency. A set of 120, 67, and 56 BC_1_F_5_ plants were generated from the HAN × WTR-1 (DP-1), CH448 × WTR-1 (DP-4), and Z413 × WTR-1 (DP-7) cross combinations to make a total of 243 BC_1_F_5_ plants. The parental lines and their MPs along with four checks (Rc 222, SL8, Rc 192, and PSB Rc 82) for each trait underwent phenotypic evaluation to assess the genetic variation and dissect the locus conferring the FATs at DGI stages of fertilizer application under three different N, P, and K fertilizer doses.

### Experimental layout

The introgression lines and their parental lines were seeded on the experimental farm at IRRI, Los Baños, Philippines (14°11N, 121°15E), during the wet season of 2017. A total of six individual plots separated by ridges were modified in a grid to maintain two replications. An alpha lattice experimental design was followed to analyze the experimental significance of the laid-out experiment. Each plot size was 245.76 m^2^ and each entry was planted in 0.4-m rows, with 0.2-m distance between rows and space of 0.2 m between adjacent plants. The 400-μm-thick transparent polyethylene sheets were inserted into the soil to cover the individual plots. At 21 days after seeding, seedlings were transplanted in an area of 491.52 m^2^ in two replications. The whole experiment was laid out with three different rates of N, P, and K fertilizer. The first rate of fertilizer consisted of complete N, P, and K that is also a recommended dose of fertilizer designated as 100% (N-P_2_O_5_-K_2_O = 100:30:30 kg ha^-1^); the second rate is a suboptimal dose, designated as 80% (N-P_2_O_5_-K_2_O = 80:30:30 kg ha^-1^); and the third rate is zero fertilizer dose, designated as 0% (N-P_2_O_5_-K_2_O = 0:0:0 kg ha^-1^). Furthermore, during crop growth, the fertilizers were applied in four DGI at 5, 20, 35, and 50 days after transplanting (DAT). Table 1 presents the detailed DGI at different fertilizer doses.

**Table 1:** Time and doses of fertilizer applications during the experiment.

Time of fertilizer application	Type of fertilizer			Fertilizer
	NH_4_NO_3_	NaH_2_PO_4_.2H_2_O	K_2_SO_4_	(kg ha^‒1^)
Recommended dose (100%)				
5 DAT^a^ (basal)	30	30	30	183.34
20 DAT	30	-	-	66.67
35 DAT	30	-	-	66.67
50 DAT	10	-	-	22.22
Total	100	30	30	338.90
Suboptimal dose (80%)				
5 DAT (basal)	24	24	24	160.00
20 DAT	23	-	-	44.44
35 DAT	23	-	-	44.44
50 DAT	10	-	-	22.22
Total	80	24	24	271.10

^**a**^Days after transplanting.

### Phenotypic characterization of the MPs for FATs

The parental lines and their MPs along with checks were evaluated for the key traits PH, TN, and LC. The trait PH was measured from the soil surface to the longest tip of the leaf within a hill. TN was recorded as shoots with at least one noticeable leaf calculated as a tiller. The main stem tiller was counted as one tiller and included in the total tiller number, whereas LC was measured in the middle leaf portion by using the portable, non-destructive chlorophyll meter SPAD-502Plus (Konica Minolta, Japan). The mean LC value indicates the relative chlorophyll content. The phenotypic data were collected from three plants selected from the middle of each entry at four DGI stages every 15 days after fertilizer application. The first fertilizer application was done at 5 DAT and was designated as stage-I. Similarly, stage-II, stage-III, and stage-IV DGIs were designated for fertilizers applied at 21 DAT, 36 DAT, and 51 DAT. The phenotypic data were expressed as mean values for each of the observations recorded. The mean values were further used for the localization of genomic regions conferring these traits.

### Genotyping

**Fig 1:**
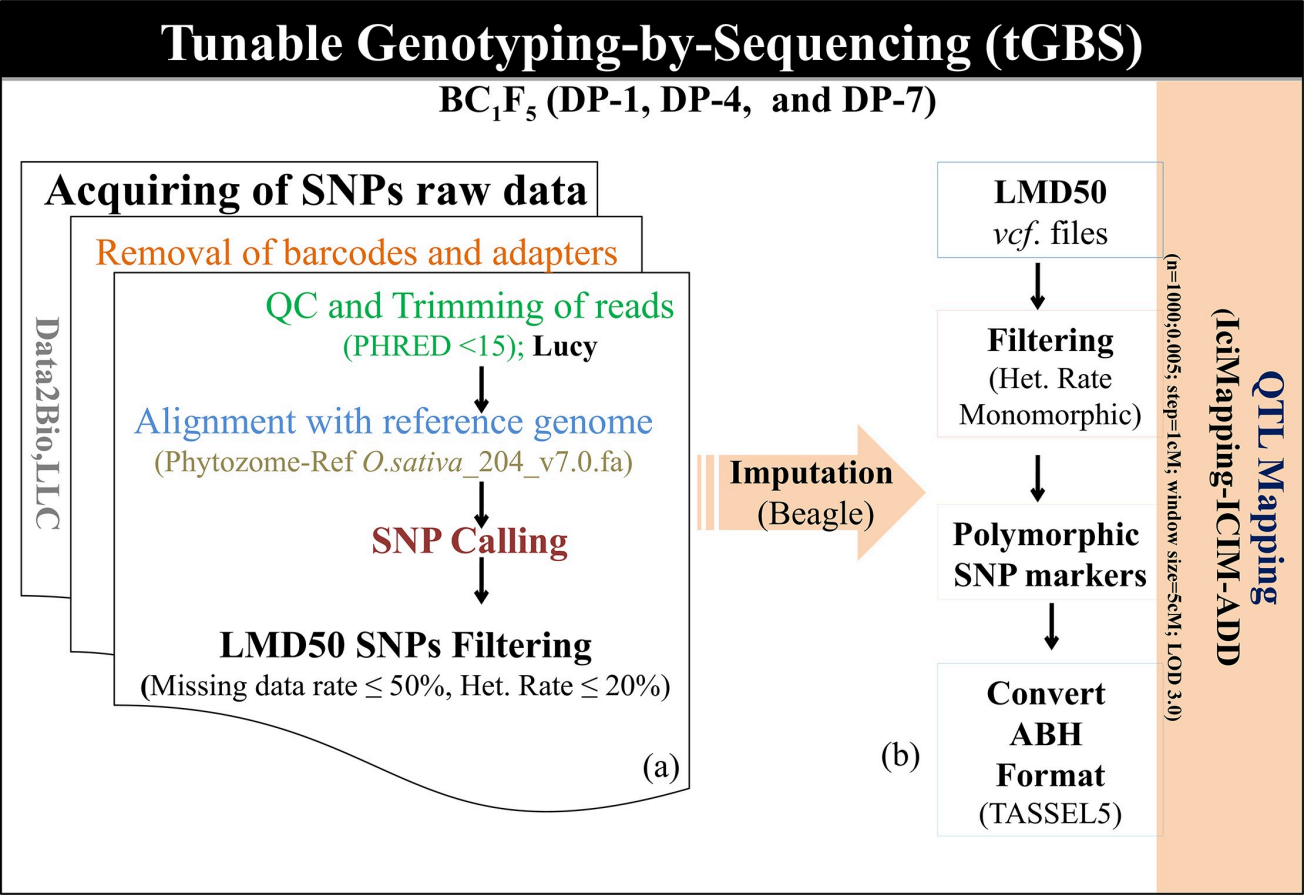
A diagramatic representation of the work-flow of tGBS data analysis. (a) Trimming of nucleotide raw sequence reads, SNP discovery, SNP calling, and removal of low-quality SNPs were performed by Ali et al. [73]. (b) The LMD50 SNP files (low missing SNP data rate of ≤50%) of three genetic backgrounds (DP-1, DP-4, and DP-7 MPs) were filtered SNPs and converted to ABH format for QTL mapping studies.

Genomic DNA was extracted from individual plants of the MPs along with the parental lines using the DNeasy Plant Mini Kit (Quiagen, USA). The quality and quantity of the isolated DNA were analyzed using a Nanodrop 2000 spectrophotometer (Thermo Fisher Scientific, Waltham, MA) and sent to Data2Bio for tGBS analysis, in which all samples were sequenced through 10 Ion Proton runs. Further, SNP calling and filtration of the generated sequencing information were processed by adopting the methodology developed by Data2Bio, LLC (https://www.data2bio.com), as described by Ali et al. [73]. For the SNP filtration, the threshold of 50% missing rate (LMD50 = minimum calling rate) was set to filter out SNPs having more than 50% missing rate across the MPs. The missing SNPs that were showing ≤ threshold missing rate were imputed using Beagle software ver. 3.3.2 [75]. Additionally, another level of SNP filtering was performed to remove mono and hetero SNPs for the individuals of the MPs using TASSEL ver. 5 and finally exported as HapMap format. The obtained HapMap format SNP marker file was again processed in MS-Excel ver. 2010 to prepare an input file for the QTL analysis [76]. The complete work-flow of tunable GBS data analysis and its use for QTL mapping studies are represented in Fig 1.

### Construction of linkage map and QTL analysis

The inclusive composite interval mapping (ICIM-ADD) method is available in QTL IciMapping ver. 4.1 software [77] for scanning the FAT data at DGI stages from three cross combinations with different nutrient fertilizer doses (0%, 80%, and 100% N, P, and K fertilizer). Before construction of the linkage map, SNPs were analyzed for segregation distortion using the Chi-square test at a significance of *P* = 0.05. The SNPs that were showing significant segregation distortion and were co-located in the same physical position were filtered out using the redundant marker removal feature of the software. The processed high-quality SNP markers were used for constructing the genetic map to assign markers on each linkage group. Kosambi mapping function was used to compute the genetic distance using recombination fraction (cM) [78]. The precisely estimated phenotypic data of FATs at different DGIs for each nutrient dose (0%, 80%, and 100% NPK) for each cross combination and high-resolution SNP marker data were used for the QTL analysis. In order to identify the QTLs with high precision and to declare significant QTLs, a 1000 permutation test at the 0.01 level of significance was considered. The linkage map and QTL mapping were constructed for each MP derived from each cross combination (DP-1, DP-4, and DP-7) separately. The detected QTLs which showed >10.00% R^2^ value were considered as major QTLs.

### Candidate gene analysis

To identify the candidate genes within the QTLs, the interval region of both flanking marker positions was considered. The physical position of the SNP flanking markers was used to determine the size of each QTL in kb. Publically accessible MSU Rice Genome Annotation Project (Osa1) release 7 (http://rice.plantbiology.msu.edu/) and the Rice Annotation Project (https://rapdb.dna.affrc.go.jp/) were explored to select the candidate genes residing in each detected QTL. Further, *in silico* gene expression analysis carried out using the genome wide expression-profiling database, RiceXPro (http://ricexpro.dna.affrc.go.jp). This is the repository of the microarray profiling data of Nipponbare cultivar isolated from different growth and developmental stages.

### Statistical analysis

The phenotypic data of each trait at four DGI stages for the three fertilizer doses were analyzed using analysis of variance (ANOVA) for assessing the significance of the experiments at the level of significance *P* = 0.05. DMRT and Fisher’s t-test were used to ascertain the significant difference between the genotypes and to compare with the check values. The correlation between the traits at different fertilizer doses (0%, 80%, and 100% N, P, and K) was computed by Pearson’s correlation analysis and the correlation matrix was visualized using *corrplot* package in R (https://github.com/taiyun/corrplot) [79]. The phenotypic mean values of all the traits were prepared using MS-Excel ver. 2010 and the analysis was carried out using PBTools (Version 1.4, http://bbi.irri.org/products). Chi-square goodness of fit was used to analyze the segregation pattern of SNP markers before using these for the QTL analysis. The multiple regression model of maximum likelihood was employed for the composite interval mapping.

## Results

### The genetic variation of phenotypic traits among different MPs

The early backcross MPs derived from three different genetic backgrounds were evaluated for three FATs under 100% NPK (338.90 kg ha^‒1^), 80% NPK (271.90 kg ha^‒1^), and 0% NPK (0 kg ha^‒1^) treatments at four DGI stages (I, II, III, and IV). The segregation pattern of FATs was determined based on the skewness and kurtosis values. For the MP DP-1, normal distribution was observed for the trait PH at stage II under 100% fertilizer rate; at stages I, II, and III at 80% fertilizer rate; and at stages I and II at 0% fertilizer rate. For the MPs DP-4 and DP-7, most of the traits at different stages and different fertilizer rates showed normal distribution (Table 2 and S1 Fig). ANOVA showed significant variation existing in the three concentrations of N, P, and K fertilizers at specific growth stages for each trait. The F-test statistic values showed the existence of significant variation at the level of significance *P* = 0.05, indicating a large amount of genetic variation among the MPs (Table 3). Among the MPs, DP-7 exhibited the highest PH at two stages of PH-I and IV (40.51, 92.20 cm), TN at stage IV (43.22 cm), and for LC at two stages, LC-II and LC-IV (44.55 and 45.71 cm, respectively), compared with the other donor and recurrent parents under low input (0% NPK) conditions. The coefficient of variation (CV) revealed the extent of phenotypic variation existing among the different traits for all of the genetic backgrounds of ILs evaluated at DGI and different doses of fertilizer. The highest CVs were observed for TN-III (37.58%), TN-IV (21.82%), and TN-IV (22.90%) in DP-1, DP-4, and DP-7, respectively, under 100% NPK conditions. In contrast, in 80% NPK conditions, TN-IV (32.47% and 38.36%) in DP-1 and DP-4, respectively, and TN-I (22.60%) in DP-7 showed the highest CVs. Under 0% NPK conditions, the highest CV was observed for trait TN at stage III (39.17% and 40.94%) in MPs DP4 and DP7, respectively, followed by CV of 32.84% at stage II in MP DP-1. As expected, the lowest CV values were recorded in the second stage of LC in DP-1, DP-4, and DP-7 in all the different doses of fertilizer. The broad-sense heritability (*H*^*2*^_*b*_) was estimated for each trait under different doses of NPK fertilizers at DGI stages. Under 100%, *H*^*2*^_*b*_ ranged from 15% to 51%. The highest *H*^*2*^_*b*_ was noted in TN-IV (51%), followed by LC-II and PH-IV, being the same estimated value of 46%. Similarly, in 80% and 0% NPK conditions, *H*^*2*^_*b*_ ranged from 10% to 65% and 6% to 65%, respectively.

**Table 2:** Descriptive statistics of three FATs at DGI stages in three different MPs.

Trait and stage of application of fertilizer	FD^a^	HAN × WTR-1					CH448 × WTR-1					Z413 ×WTR-1				
		Mean±SD^b^	Range	CV^c^ac	SK^d^	KT^e^	Mean±SD^b^	Range	CV^c^	SK^d^	KT^e^	Mean±SD^b^	Range	CV^c^	SK^d^	KT^e^
PH-I (5 DAT)	100%	40.49±4.04	32.00–55.70	9.98	0.45	0.25	40.09±2.87	34.25–48.17	7.15	0.48	-0.18	40.14±3.26	27.01–47.17	8.12	-0.88	2.61
PH-II (20 DAT)		64.81±6.80	49.53–80.83	10.49	0.06	-0.81	62.64±5.23	52.73–76.23	8.35	0.13	-0.29	62.21±5.07	47.41–72.67	8.15	-0.68	0.35
PH-III (35 DAT)		83.05±7.66	60.67–105.00	9.22	0.14	0.09	78.09±7.39	54.83–97.93	9.46	-0.07	0.21	77.92±6.49	62.63–93.47	8.32	-0.39	-0.18
PH-IV (50 DAT)		93.89±9.49	67.33–122.33	10.11	0.41	0.56	86.54±8.16	68.67–107.01	9.43	0.25	-0.21	87.12±6.12	76.50–106.25	7.02	0.63	0.56
TN-I (5 DAT)		5.58±1.48	1.33–11.00	26.52	0.50	0.49	5.05±1.02	2.67–7.67	20.30	0.24	-0.04	5.27±1.12	2.01–8.11	21.49	0.62	0.74
TN-II (20 DAT)		11.46±2.12	6.33–17.67	18.50	0.32	-0.02	15.61±26.38	7.45–287.83	9.01	6.82	81.02	12.53±1.96	7.50–18.01	15.64	0.44	0.01
TN-III (35 DAT)		15.91±5.98	7.33–48.33	37.58	2.72	9.76	14.15±2.61	10.87–24.33	18.46	1.19	2.21	14.31±2.74	8.67–23.50	19.15	0.65	0.88
TN-IV (50 DAT)		16.11±4.28	10.00–34.33	26.58	1.47	2.95	15.14±3.30	8.67–25.56	21.82	0.48	0.07	15.23±3.50	8.67–26.33	22.98	0.84	1.32
LC-I (5 DAT)		41.16±2.80	33.03–48.00	6.80	-0.45	0.06	40.68±2.95	31.03–47.53	7.26	-0.36	0.48	43.27±2.75	34.70–48.43	6.36	-0.69	0.48
LC-II (20 DAT)		42.92±2.14	37.17–48.90	4.99	-0.31	-0.19	42.97±2.51	36.68–48.63	5.83	-0.03	-0.13	44.75±2.27	38.37–53.57	5.07	0.11	2.31
LC-III (35 DAT)		43.33±2.82	33.67–51.33	6.51	-0.35	0.29	39.76±3.60	30.57–49.27	9.05	0.10	0.01	41.12±3.22	32.37–48.27	7.83	-0.22	-0.26
LC-IV (50 DAT)		44.21±3.40	30.17–54.80	7.69	-0.55	1.81	40.78±4.00	26.58–54.97	9.81	0.01	1.72	42.81±3.63	33.15–50.47	8.48	-0.44	-0.38
PH-I (5 DAT)	80%	39.55±3.64	29.00–52.10	9.20	0.07	0.24	38.92±3.79	29.01–51.01	9.73	0.02	0.45	39.94±3.58	31.73–48.01	8.97	-0.27	-0.53
PH-II (20 DAT)		64.98±6.41	48.30–84.00	9.86	-0.04	0.21	65.03±5.70	55.33–81.30	8.77	0.83	0.24	65.85±5.21	51.71–76.70	7.92	-0.4	0.01
PH-III (35 DAT)		76.59±7.93	57.00–105.00	10.35	0.10	0.30	75.95±7.80	60.24–97.78	10.27	0.34	0.12	77.60±6.86	62.71–92.70	8.84	-0.15	-0.56
PH-IV (50 DAT)		87.81±9.30	65.70–120.70	10.59	0.39	0.61	85.61±8.08	67.23–105.49	9.44	0.11	-0.32	87.48±7.85	57.72–105.01	8.98	-0.58	1.53
TN-I (5 DAT)		5.13±1.04	2.30–8.70	20.29	0.40	0.51	4.83±1.04	2.87–8.45	21.47	0.07	1.28	5.23±1.19	3.34–10.11	22.66	0.88	2.02
TN-II (20 DAT)		11.54±2.21	6.30–19.70	19.18	0.27	0.23	11.07±2.19	6.70–17.00	19.83	0.15	-0.48	12.56±2.55	7.00–18.30	21.26	0.43	-0.01
TN-III (35 DAT)		15.82±3.70	9.00–30.00	23.35	1.05	1.79	15.03±3.15	6.07–24.05	20.94	0.22	1.00	15.78±3.25	9.70–26.70	20.62	0.93	1.06
TN-IV (50 DAT)		16.06±5.21	8.30–50.00	32.47	2.80	13.93	15.58±5.98	9.30–65.00	38.36	5.38	40.97	15.79±3.45	9.11–28.01	21.86	0.81	1.37
LC-I (5 DAT)		41.35±3.53	27.60–53.10	8.53	-0.68	1.74	40.60±3.27	31.50–47.40	8.06	-0.49	0.17	42.68±3.77	30.5–49.30	8.84	-1.09	1.68
LC-II (20 DAT)		42.50±3.45	29.90–77.10	8.13	4.44	46.70	41.91±2.76	32.70–47.90	6.59	-0.84	1.27	44.11±2.19	37.4–48.40	4.97	-0.29	-0.04
LC-III (35 DAT)		40.77±3.33	21.50–48.30	8.17	-1.20	5.33	39.54±4.02	29.30–49.90	10.16	-0.24	-0.19	41.64±3.63	30.3–54.10	8.71	-0.1	1.11
LC-IV (50 DAT)		41.76±3.80	28.70–61.90	9.09	0.30	3.50	40.92±3.44	33.10–48.60	8.41	-0.18	-0.75	42.87±2.99	33.6–47.70	6.97	-0.77	0.41
PH-I (5 DAT)	0%	39.85±3.06	25.83–49.23	7.69	0.01	1.67	40.46±3.23	32.70–50.59	7.99	0.32	0.62	40.51±3.61	32.17–49.83	8.92	-0.15	-0.05
PH-II (20 DAT)		61.41±5.74	46.90–74.93	9.35	0.01	-0.22	67.43±14.14	50.20–205.30	20.97	8.09	78.98	64.97±6.58	48.57–83.37	10.13	-0.25	0.27
PH-III (35 DAT)		77.06±7.32	51.83–95.60	9.50	-0.25	0.08	82.28±7.63	62.33–100.67	9.27	0.12	-0.03	81.66±7.63	61.17–98.67	9.34	-0.14	-0.28
PH-IV (50 DAT)		86.78±8.10	56.12–112.00	9.33	-0.14	0.79	92.09±8.70	67.33–117.33	9.45	0.21	0.32	92.20±8.17	77.67–117.67	8.86	0.74	0.73
TN-I (5 DAT)		4.99±1.21	1.33–12.00	24.22	0.94	5.41	5.60±1.31	2.67–9.67	23.39	0.57	0.54	5.44±1.32	2.67–8.33	24.25	0.19	-0.35
TN-II (20 DAT)		12.61±4.14	6.50–65.00	32.84	9.62	120.16	11.52±2.16	6.67–17.33	18.73	0.11	-0.09	11.06±2.04	7.11–16.33	18.43	0.25	-0.18
TN-III (35 DAT)		14.48±4.68	9.33–55.67	32.30	5.51	42.99	16.46±6.45	8.00–46.33	39.17	2.74	8.79	15.97±6.54	8.67–47.14	40.94	2.84	9.78
TN-IV (50 DAT)		14.97±3.69	8.33–40.00	24.66	1.85	8.84	16.26±3.91	7.67–31.33	24.07	0.94	1.37	16.03±5.56	9.33–43.67	34.69	2.16	7.08
LC-I (5 DAT)		41.28±2.40	34.13–46.50	5.80	-0.21	-0.14	40.99±2.96	30.07–47.97	7.22	-0.58	1.42	43.22±2.95	35.86–49.13	6.83	-0.42	-0.24
LC-II (20 DAT)		43.62±2.24	37.10–48.80	5.15	-0.36	0.18	43.05±2.33	37.83–49.23	5.41	0.11	-0.35	44.55±2.35	34.53–50.43	5.28	-0.77	3.11
LC-III (35 DAT)		40.11±3.83	28.93–48.10	9.55	-0.46	-0.06	42.59±3.19	33.40–48.80	7.52	-0.51	-0.04	44.44±2.74	37.93–50.87	6.17	-0.28	-0.39
LC-IV (50 DAT)		41.92±3.23	34.20–50.05	7.70	-0.19	-0.17	43.58±3.36	33.93–54.50	7.71	-0.02	0.41	45.71±3.75	37.37–59.60	8.21	1.11	2.85

^**a**^Fertilizer dose

^**b**^Standard deviation

^c^Coefficient of variation

^d^Skewness

^e^Kurtosis

**Table 3:** Analysis of variance (ANOVA) and its components for all the FATs in DGI stages under different doses of fertilizer.

Fertilizer dose	Components	PH-I	TN-I	LC-I	PH-II	TN-II	LC-II	PH-III	TN-III	LC-III	PH-IV	TN-IV	LC-IV
100%	DF	243.00	243.00	243.00	243.00	243.00	243.00	243.00	243.00	243.00	243.00	243.00	243.00
	SS	4302.66	370.93	2537.87	10817.20	1060.06	1501.87	16348.90	7816.03	2099.75	23329.00	5355.86	2960.64
	MS	20.49	1.77	12.09	51.5	5.05	7.15	77.85	37.22	10.00	111.09	25.50	14.10
	F value	1.49	1.17	1.87	1.70	1.41	1.86	1.64	1.28	1.23	1.85	2.06	1.37
	Pr (>F)	0.00	0.13	0.00	0.00	0.01	0.00	0.00	0.04	0.07	0.00	0.00	0.01
	*H*^*2*^_*b*_ (%)	0.33	0.15	0.46	0.41	0.29	0.46	0.39	0.23	0.20	0.46	0.51	0.27
80%	DF	243.00	243.00	243.00	243.00	243.00	243.00	243.00	243.00	243.00	243.00	243.00	243.00
	SS	3490.61	255.74	2924.77	10295.80	1218.81	2383.19	16584.60	3332.20	2807.13	21903.40	4973.05	3191.20
	MS	16.70	1.22	13.99	49.26	5.83	11.40	79.35	15.94	13.43	104.80	23.79	15.27
	F value	1.75	1.43	1.33	2.16	1.51	1.31	2.31	1.41	0.97	2.83	1.32	1.47
	Pr (>F)	0.00	0.01	0.02	0.00	0.00	0.02	0.00	0.01	0.60	0.00	0.02	0.00
	*H*^*2*^_*b*_ (%)	0.43	0.30	0.26	0.54	0.34	0.24	0.57	0.31	0.10	0.65	0.24	0.33
0%	DF	243.00	243.00	243.00	243.00	243.00	243.00	243.00	243.00	243.00	243.00	243.00	243.00
	SS	2619.52	899.32	1922.82	7728.10	862.94	1656.49	16616.30	2971.73	3131.77	19960.60	3029.76	3168.06
	MS	12.53	4.30	9.20	36.93	4.13	7.93	79.50	14.22	14.98	95.51	14.50	15.16
	F value	2.13	1.07	1.61	2.05	1.27	1.91	2.37	1.13	1.21	2.89	1.95	1.37
	Pr (>F)	0.00	0.30	0.00	0.00	0.04	0.00	0.00	0.19	0.08	0.00	0.00	0.01
	*H*^*2*^_*b*_ (%)	0.53	0.06	0.38	0.51	0.21	0.48	0.58	0.11	0.20	0.65	0.49	0.27

**SV =** Source of variation; **DF =** Degrees of freedom; **SS =** Sum of squares; **MS =** Mean sum of square; **Pr =** Probability value-; **F** value- Significance level; ***H***^***2***^_***b***_ **(%) =** Broad-sense heritability.

### Pearson correlation matrix (PCM) analysis

**Fig 2:**
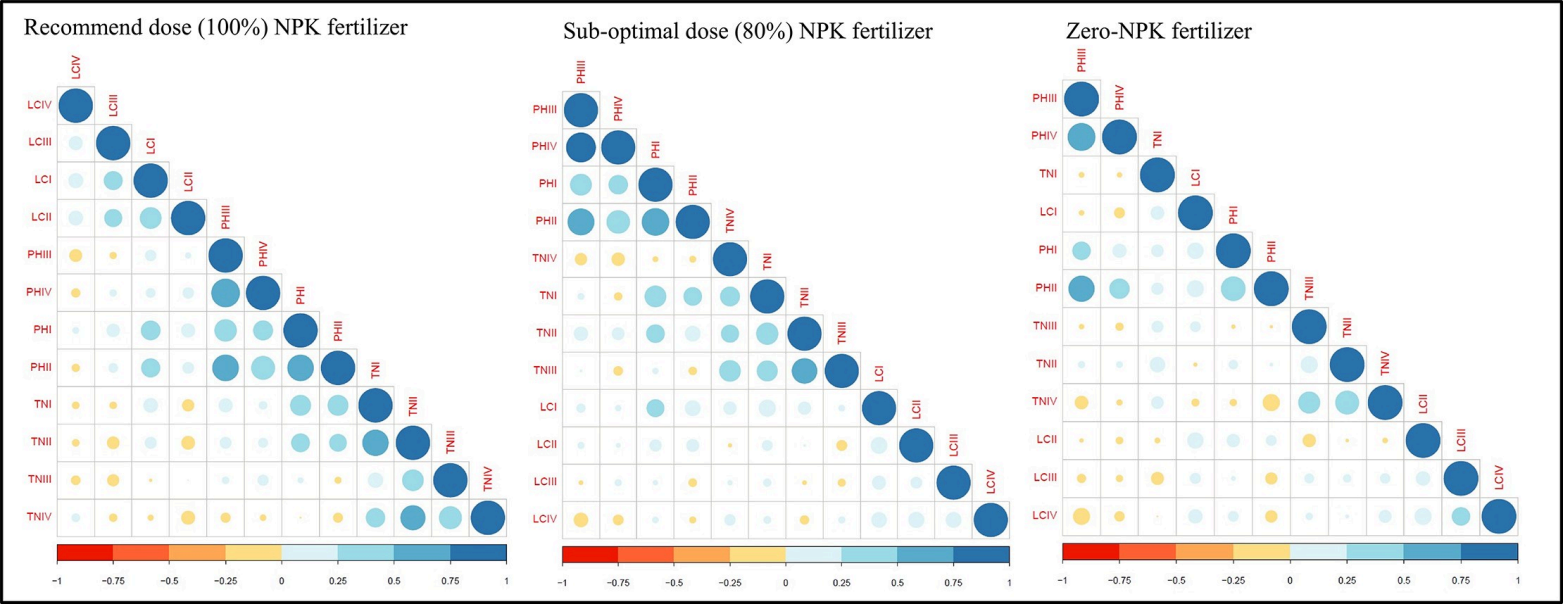
Heat map showing the PCM of FATs measured in response to different doses of N, P, and K fertilizer at DGI stages. **PH =** Plant height; **TN =** Tiller number; **LC =** Leaf chlorophyll content.

The PCM was performed in DGI stages in three genetic backgrounds of MPs to assess the correlation among the FATs (PH, LC, and TN). Most of the FATs were found to be significantly positively correlated with each other. The highest significant positive correlation was observed between PH-III and PH-IV (r = 0.68) in 100% N, P, and K. Interestingly, in 80% and 0% NPK conditions, there was no significant positive correlation between the traits. The trait LC-II showed the highest negative correlation with TN-II (r = ‒0.15) and TN-IV (r = ‒0.15) in 100% NPK conditions, followed by LC-IV with PH-III (r = ‒0.17) at 80% NPK, and PH-II with TN-IV (r = ‒0.23) at 0% NPK, as presented in Fig 2.

### SNP discovery and construction of saturated linkage map

**Fig 3:**
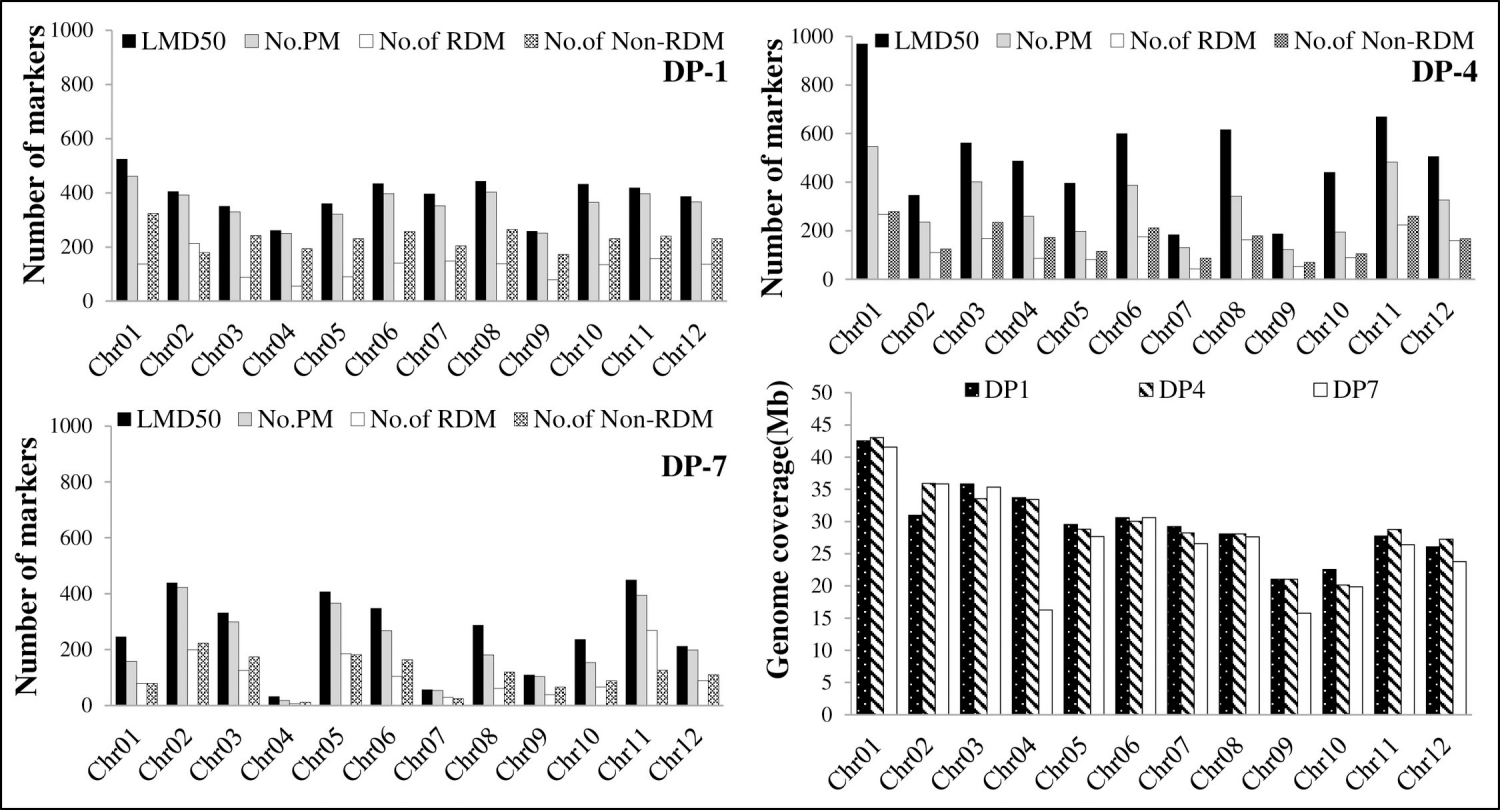
Chromosome-wise distribution of SNP markers in three populations (DP-1, DP-4, and DP-7) extracted from novel tGBS genotyping platform.

All the genotypes, including parental lines, underwent genotyping by using the advanced and cost-effective genotyping platform tGBS. It was used for the extraction of SNP markers for the localization of the genetic factors influencing FATs under different fertilizer doses. The genotyping platform tGBS yielded extensive sequence information from sequencing three MPs. A total of 10,432, 14,117, and 7,865 raw reads were obtained from the MPs as DP-1, DP-4, and DP-7, respectively (Table 4). A novel SNP calling and filtering method was followed to eliminate missing values (≥50 missing data, LMD 50) from the tunable GBS pipeline (Data2Bio, LLC) (https://www.data2bio.com), as reported in Ali et al. [73]. In the initial SNP filtering, as many as 4,666, 5,957, and 3,147 SNPs were isolated from MPs DP-1, DP-4, and DP-7, respectively, whereas, in the next level of filtering, 4,286 SNPs from DP-1, 3,622 SNPs from DP-4, and 2,610 SNPs from DP-7 were isolated (Table 4). Notably, out of these SNP markers, 35.10% in DP-1, 44.64% in DP-4, and 47.67% in DP-7 were redundant and excluded before further analysis. Finally, the SNPs were filtered out at the final level of filtering based on the segregation distortion regions in the genetic linkage map. A total of 57.80% of the SNPs demonstrated genetic distortion (*P =* 0.05) in the DP-1 population, followed by 44.63% in DP-4 and 38.72% in DP-7. The SNP markers obtained from all the filtration levels were used for linkage map construction and linkage analysis. In total, a 953.71 cM chromosomal spanning region was covered by 2,782 SNPs in DP-1, 1,398.42 cM by 2,005 SNPs in DP-4, and 834.11 cM by 1,361 SNPs in DP-7. In each population, an average of 230, 167, and 113 SNP markers were distributed across all chromosomes. The highest number of polymorphic markers was present on chromosome 1 (324 SNPs) while the lowest was on chromosome 9 (172) in DP-1, whereas, in DP-4 and DP-7, the highest number of markers was located on chromosome 1 (279) and chromosome 2 (223). However, chromosome 9 (70) and chromosome 4 (11) had the lowest number of polymorphic markers available in DP-4 and DP-7, respectively (Table 5 and Fig 3). The average distance of each SNP marker varied from 0.13 to 0.41 Mb, whereas the longest distance recorded was 1.47 Mb on chromosome 4 in the population DP-7.

**Table 4:** Processing SNP markers for QTL analysis.

S. no.	SNP calling	MPs		
		HAN × WTR-1	CH448 × WTR-1	Z413 × WTR-1
1	Raw SNPs	10,432	14,117	7,865
2	Quality base filtering at LMD50	4,666	5,957	3,147
3	Removed heterozygous alleles in both parents	345	483	454
4	Removed mono-morphic alleles in both parents	35	1,852	83
5	SNP markers for QTL analysis	4,286	3,622	2,610
6	Exclusion of redundant markers	1,504	1,617	1,249
7	Number of markers used for linkage map construction	2,782	2,005	1,361
8	Removed markers with χ^2^ >6.0 of segregation distortion	1,608	895	527
9	Number of SNP markers used for ICIM-ADD	1,174	1,110	834

**Table 5:** Distribution of SNP markers, coverage, and position of each chromosome.

Chr	HAN × WTR-1			CH448 × WTR-1			Z413 × WTR-1		
	NSM	CL (Mb)	ADSM (Mb)	NSM	CL (Mb)	ADSM (Mb)	NSM	CL (Mb)	ADSM (Mb)
1	324	42.53	0.13	279	43.03	0.15	79	41.56	0.52
2	179	30.97	0.17	125	35.90	0.29	223	35.85	0.16
3	242	35.83	0.15	234	33.55	0.14	174	35.35	0.20
4	194	33.72	0.17	173	33.41	0.19	11	16.27	1.47
5	231	29.54	0.13	115	28.81	0.25	181	27.66	0.15
6	257	30.61	0.12	212	30.04	0.14	163	30.61	0.18
7	216	29.21	0.14	87	28.25	0.32	24	26.57	1.10
8	265	28.07	0.11	179	28.05	0.16	119	27.61	0.23
9	172	21.04	0.12	70	21.03	0.30	65	15.77	0.24
10	231	22.57	0.10	105	20.12	0.19	88	19.87	0.22
11	240	27.75	0.12	259	28.76	0.11	124	26.38	0.20
12	231	26.08	0.11	167	27.26	0.16	110	23.77	0.21
Avg.	230.83	29.82	0.13	167.08	29.85	0.20	113.41	27.27	0.41
Total	2782.00	357.92	1.57	2005.00	388.06	2.41	1361.00	327.27	4.94

**Chr =** Chromosome; **NSM** = Number of SNP markers; **CL** = Chromosome length; **ADSM** = Average distance of SNP markers

### Molecular mapping of genomic regions conferring FATs

In order to locate the QTLs influencing the traits PH, TN, and LC at DGI stages under different fertilizer doses, QTL analysis was performed using the ICIM-ADD methodology. A total of 19 main-effect QTLs (M-QTLs) were identified using the composite interval mapping model of QTL IciMapping under LOD threshold of 3.00. The significant QTLs were identified on chromosomes 1, 9, 10, and 12 in 100% NPK conditions; on chromosomes 1, 2, 3, 5, and 12 in 80% NPK conditions; and on chromosomes 2, 3, 4, 5, 6, 8, and 11 in 0% NPK conditions. The detected M-QTLs explained phenotypic variation ranges from 1.89% to 34.85% with LOD score ranges from 3.02 to 28.70. The number of QTLs per trait ranged from one to four, and the highest numbers of QTLs (four QTLs) were associated with LC-II in populations DP-1, DP-4, and DP-7 (S1 Table). A total of four QTLs were discovered for LC-II, TN-I and II, and PH-III in 100% NPK; six QTLs for PH-I, III, and IV, TN-III, and LC-II in 80% NPK; and nine QTLs for PH-I, LC-I and II, and TN-II, III, and IV in 0% of NPK conditions at DGI stages. The highest number of QTLs (nine QTLs) was detected in 0% of NPK conditions, followed by 80% NPK (six QTLs) and 100% NPK (four QTLs). The majority of the negative additive effect of the QTLs was contributed by the donor parental allele source. Among the total QTLs, 57.89% (11 QTLs) were contributed by donor alleles from HAN, CH448, and Z143, whereas 42.10% came from recurrent parent WTR-1. Two M-QTLs (*qLC-II_1* and *qLC-II_11*) contributed highest to the PVE which ranged from 30.81% to 38.22% in DP-7. In all three NPK conditions, five QTLs (four QTLs for LC-II and one QTL for LC-I) were significantly associated with a single trait of LC on chromosomes 1, 2, 8, and 11. The negative additive effect of *qLC-II_80%_1* was contributed by Z143, whereas four other QTLs (*qLC-II_100%_1*, *qLC-I_0%_2*, *qLC-II_0%_8*, and *qLC-II_0%_11*) were contributed by a positive additive effect from WTR-1.

### Putative QTLs with high statistical power

**Fig 4:**
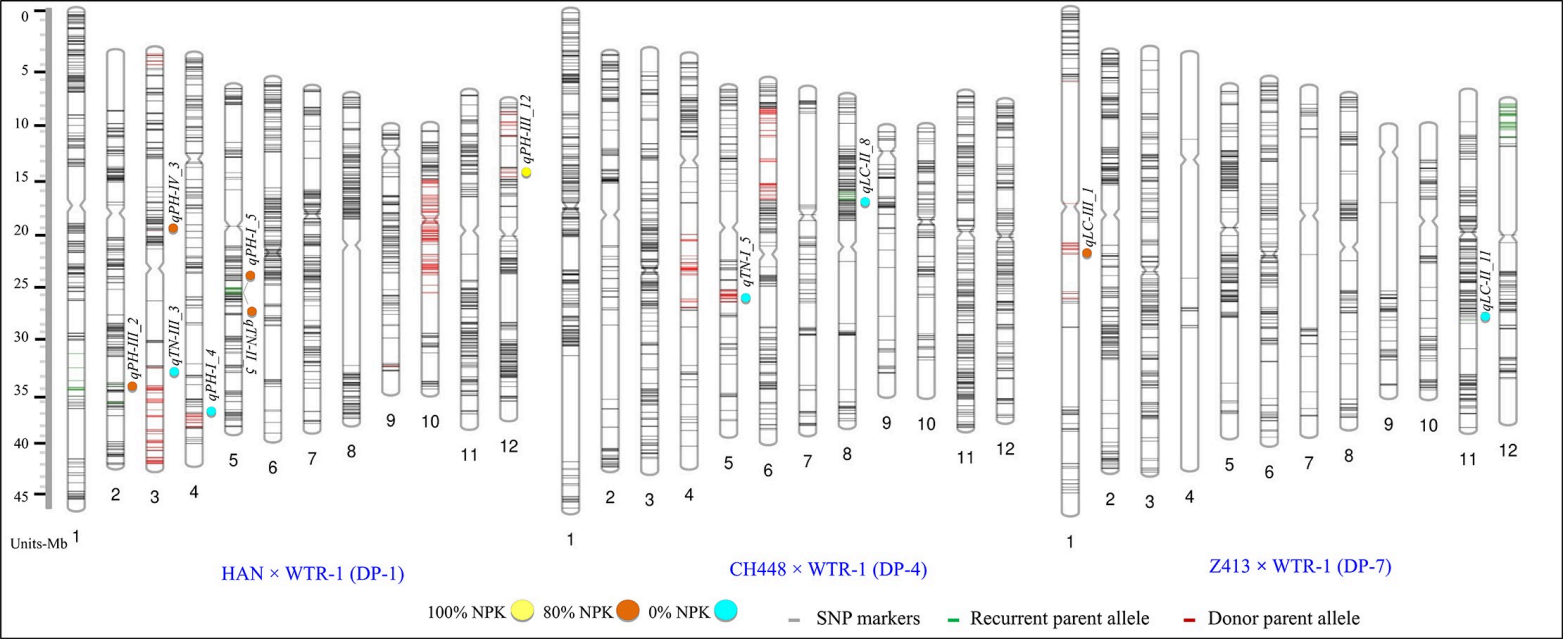
The genetic map representing the distribution of polymorphic SNP markers. Colored circles yellow, orange, and blue indicate 100%, 80%, and 0% nutrient fertilizer doses, respectively. The QTLs highlighted on the genetic map are the major QTLs detected in the study possessing ≥15% phenotypic variation explained. QTLs from different FATs with fertilizer doses are shown in colored circles.

To detect QTLs with higher statistical power, an extreme threshold parameter, 1000 permutation test at *P* = 0.01, was considered. A total of 11 putative QTLs (≥15% PVE) were detected for the three FATs at DGI stages under three different fertilizer doses (100%, 80%, and 0% NPK). The QTL *qPH-III_12* was the only one detected at 100% NPK for the FAT, PH at stage III on chromosome 12 between the markers S12_852867 and S12_2965504. Under suboptimal fertilizer dose, three QTLs for PH, *qPH-III_2*, *qPH-IV_3* and *qPH-I_5*,were detected on chromosome 2, 3 and 5, while another single QTL for LC (*qLC-II_1*) and TN (*qTN-III_5*), were detected on chromosome 1 and 5. Simialrly in zero fertilizer conditions, five QTLs (*qTN-III_3*, *qPH-I_4*, *qTN-I_5*, *qLC-II_8* and *qLC-II_11*) for PH, TN and LC were detected on five chromosomes 3, 4, 5, 8, and 11. However, the significant M-QTLs were identified only from MP DP-1, except four QTLs detected in the cross combination CH448 × WTR-1 (DP-4) and Z143 × WTR-1. The distribution of detected significant main-effect QTLs under three different NPK conditions is depicted in Fig 4. Additionally, minor effects QTLs were also detected for all the traits. The list of total minor effect QTLs including the information of flanking markers along with their LOD score, PVE and the additive effect of parental source of the allele are presented in S1 Table.

### Traits associated with putative QTLs detected at 100%, 80%, and 0% NPK fertilizer dosage

Across the three MPs, a single QTL (*qPH-III_12*) was identified at 100% NPK fertilizer dose for the trait PH at stage III of fertilizer application. This QTL is located on chromosome 12 and it explained 23.60% of PV. The negative additive effect is contributed by donor parent HAN. Under suboptimal conditions (80% recommended dose of fertilizer), five QTLs (*qLC-II_1*, *qPH-III_2*, *qPH-IV_3*, *qPH-1_5*, and *qTN-III_5*) were detected on three chromosomes as 1, 2 and 3. Those QTLs were associated with PH, TN, and LC at DGI stages I, II, III, and IV. Among the 11 main-effect QTLs, *qLC-II_1* showed the highest PVE of 34.85%. Except for the chromosome 1 QTL (*qLC-II_1*), all of them were identified in a single MP, DP-1 (S1 Table). Similarly, QTL analysis under zero fertilizer dose revealed that a total of five main-effect QTLs were detected for the traits of PH-I, TN-I and III, and LC-II. Three QTLs (*qTN-III_3*, *qPH-I_4*, and *qTN-I_5*) were detected on chromosomes 3, 4, and 5 and they explained PV of 17.02%, 19.17%, and 28.44% at stages I and III of fertilizer application. Another two QTLs (*qLC-II_8* and *qLC-II_11*) were associated with a single trait (LC) at first stages of fertilizer application. QTLs on chromosome 3, 4, and 5 were contributed by the negative additive effect from donor alleles, whereas QTLs on chromosomes 8 and 11 were contributed by the positive additive effect from the WTR-1 allele.

### Exploring putative QTLs for candidate genes for future breeding programs

**Fig 5:**
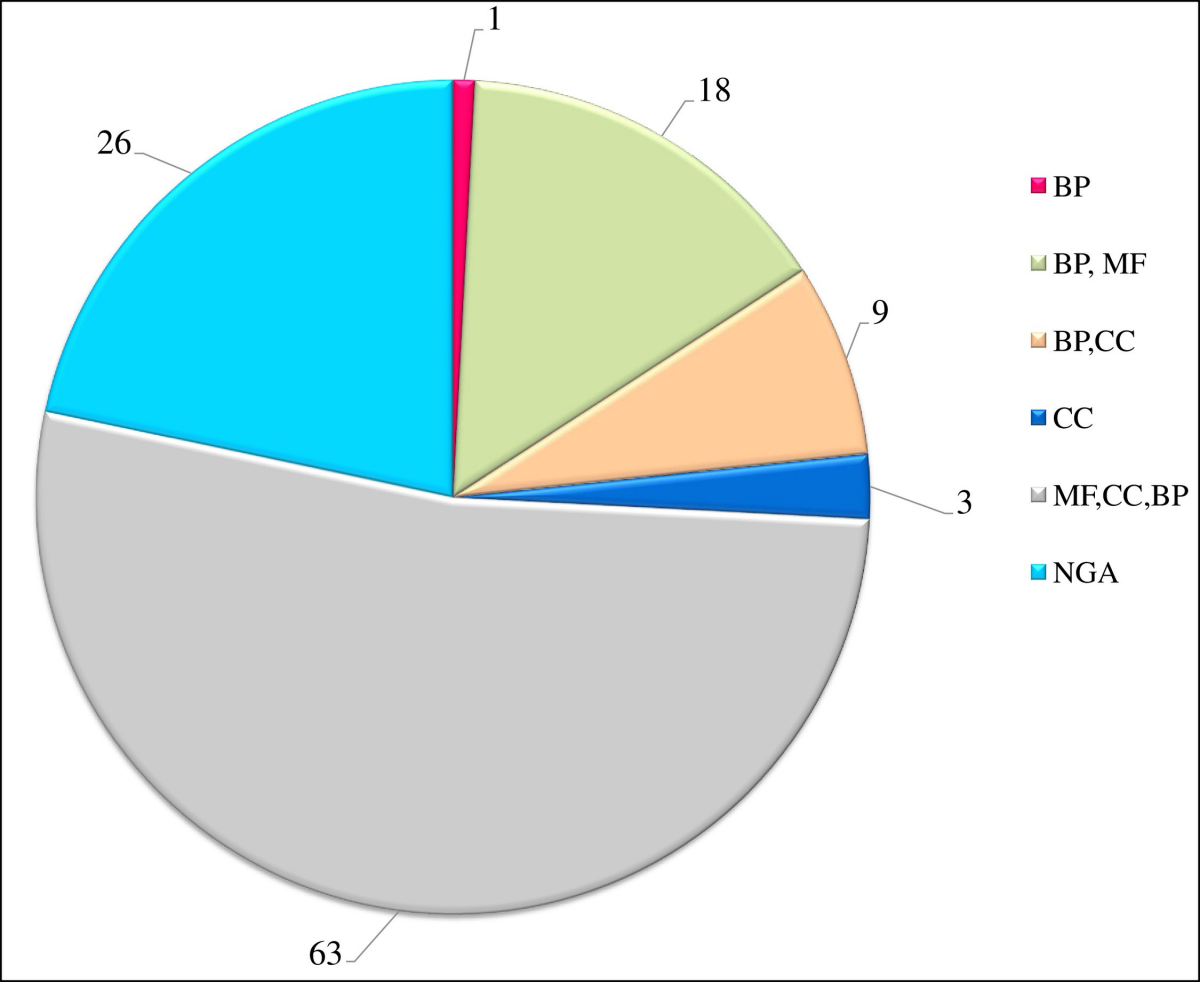
**Functional classification of annotated genes (MF =** Molecular function; **CC =** Cellular component; **BP =** Biological process; **NGO =** No gene ontology classification).

The putative QTLs detected from the extreme threshold parameters were further explored to showcase the possible candidate genes within the loci to understand the molecular and physiological mechanisms underlying the traits conferring NPK deficiency tolerance. A total of 19 M-QTLs were located on all chromosomes, except on chromosome 7. Further, the possible candidate genes within the M-QTLs were filtered based on the SNP flanking marker positions with a threshold score of ≤1 Mb interval regions of M-QTLs (Table 6). The lowest number (3) and highest number (25) of candidate genes were associated with a single trait (TN at stages III and IV) in the MP DP-1. A total of 120 genes were identified (S2 Table), of which 78.33% (94 genes) were functionally annotated while 21.66% (26 genes) were reported as hypothetical, retrotransposon proteins, and expressed proteins. The 94 functionally annotated genes were classified into six biological functions related to cellular component (CC), biological process (BP), and molecular function (MF) (Fig 5). Out of these 94 genes, 63 candidate genes were involved in MF, CC, and BP, followed by 18 genes for BP and MF, 9 genes for BP and CC, 3 genes for CC, and a single gene for BP.

**Table 6:** Putative QTLs (≤1 Mb interval regions) at DGI stages for favorable agronomic traits.

Cross combination	Fertilizer dose (% NPK)	QTL designation	Chromosome	Left marker	Right marker	Size of the QTL (kb)	LOD score	Additive effect	R^2^ (%)	No. of candidate genes detected within the locus
HAN × WTR-1	80%	*qPH-III_2*	2	S2_29072519	S2_28879120	193.40	3.65	2.86	18.12	15
HAN × WTR-1	0%	*qLC-I_2*	2	S2_30451119	S2_30564762	113.60	3.04	0.64	10.79	12
HAN × WTR-1	80%	*qPH-I_5*	5	S5_17801786	S5_17996757	195.10	3.59	1.21	15.61	16
HAN × WTR-1	80%	*qTN-III_5*	5	S5_17859957	S5_17801758	58.20	26.77	3.62	28.98	3
HAN × WTR-1	0%	*qTN-IV_5*	5	S5_17465069	S5_17801758	336.70	25.70	4.72	5.85	25
CH448 × WTR-1	0%	*qLC-II_8*	8	S8_8206216	S8_8918154	711.90	5.26	1.37	22.85	30
HAN × WTR-1	100%	*qTN-I_9*	9	S9_20844013	S9_20779092	64.92	3.11	-0.65	13.00	10
HAN × WTR-1	80%	*qTN-III_12*	12	S12_6585982	S12_5905028	681.11	3.10	-1.00	1.89	9

## Discussion

For the ever-increasing global population and to meet food demand, the development of rice varieties with higher grain yield is essential [80]. The application of the key nutrients N, P, and K either organically or through chemical fertilizer plays a foremost role in increasing yield and sustaining soil fertility [81]. With the recent trends in crop breeding, higher rice productivity has been successfully attained by applying high fertilizer doses [9,35,82,83]. However, without knowledge of the correct stage, timing, and dose of fertilizer application, any assurance for increasing yield cannot be determined. The excessive use of fertilizer is a major contributor to increasing soil, water, and environmental pollution, along with farm operation costs [9,18,33,84]. China is leading in per hectare usage of fertilizer (300 to 350 kg ha^-1^ N in Jiangsu Province), and this amount is around four times higher than the average world fertilizer consumption for rice production [18,33]. Hence, it is crucial to undertake a systematic breeding program involving identifying genotypes with higher input use efficiency and genomic regions derived from these lines to improve elite varieties to assure sustainable crop production. This would not only reduce environmental and human hazards but would also improve the livelihood of farmers by reducing farm operational costs along with a higher expected grain yield. Hence, in the present study, we have developed MPs generated from three different donors (HAN, CH448, and Z413) and evaluated them under three different fertilizer doses, 100% (222.23 kg ha^-1^ N), 80% (177.77 kg ha^-1^ N), and 0% (0 kg ha^-1^ N), to study the genetic variation for low input use efficiency, especially for N, P, and K. Further, we identified the genomic regions influencing FATs at DGI stages under different fertilizer doses while dissecting the molecular genetic information.

### Dissecting molecular genetics of nutrient deficiency tolerance in rice

ANOVA carried out using different MPs for FATs at different stages indicated the existence of significant variation among all the MPs for the target traits. As expected, all the genotypes, including parents of the MPs, showed a higher mean performance for all the AFTs at 100% NPK, followed by 80% and 0% NPK. This trend observed mainly because of the higher doses of the key nutrients N, P, and K, which are proven to enhance rice yield. Several researchers also reported the similar effect of key nutrients on plant growth and yield-attributed traits [85–88]. The variance components of FATs indicated that the segregation pattern at different DGI stages under different fertilizer doses varied from normal to skewed distribution (Table 2). This clearly confirmed the influence of few or many genes with a cumulative and additive effect, which is difficult to dissect using traditional low-resolution genotyping platforms. Hence, to understand the molecular genetic basis of the traits that influence the genomic regions conferring FATs at different DGI stages under varied fertilizer doses, a high-resolution and informative genotyping platform was employed. Numerous high-quality SNPs retrieved from the advanced genotyping tool tGBS were used for the molecular mapping of the key traits. A total of 19 M-QTLs were identified from three different MPs for the three FATs at four DGI stages under three different fertilizer doses. Out of these QTLs, 13 QTLs from DP-1, 4 QTLs from DP-4, and 2 QTLs from DP-7 explained PV ranging from 1.89% to 28.98%, 7.74% to 28.44%, and 30.62% to 34.85%, respectively. With the extreme threshold parameters, eight M-QTLs were considered as putative QTLs possess ≤1 Mb QTL regions, whereas the remaining 11 QTLs possess ≥1 Mb genetic regions of QTLs (Table 6). The putative QTLs *qPH-III_2*, *qLC-I_2*, *qPH-I_5*, *qTN-III_5*, *qTN-IV_5*, *qLC-II_8*, *qTN-I_9*, and *qTN-III_12* suggested that these are closely associated with the respective traits (PH, TN, and LC) at DGI stages of fertilizer application. However, the majority of these QTLs on chromosomes 2, 5, and 8 were attributed to WTR-1 allele, whereas the QTLs on chromosomes 9 and 12 by HAN.

### Potentiality of the putative QTLs and promising ILs

The mean performance of all the individuals of the MPs showed higher values than their parents for all the FATs (Table 2; S1 Fig). This indicates a more positive response by ILs to fertilizer application than their parents, even to suboptimal and zero fertilizer doses. The main reason for the improved performance of the ILs is the existence of transgressive segregation for all the FATs at all DGI stages under different fertilizer doses. The QTL *qTN-I_9* detected under 100% NPK was found to be responsible for the transgressive segregation of the ILs for the trait TN for the mapping population derived from DP-1. Similarly, QTLs *qPH-III_2*, *qPH-I_5*, *qTN-III_5*, and *qTN-III_12* contributed to the superior performance of ILs from the MPs derived from DP-1. Most importantly, three QTLs, *qLC-I_2*, *qTN-IV_5*, and *qLC-II_8*, detected on chromosomes 2, 5, and 8 at 0% NPK fertilizer dose, contributed solely to the better phenotypic performance of the ILs in MPs DP-1 and DP-4. This finding has an immense application in MAS through the introgression of the same QTLs in the background of elite lines to give them more NuUE under zero fertilizer conditions. To date, very few rice cultivars with tolerance of low inputs have been identified by using different doses of individual N, P, and K fertilizers reported from various phenotypic screening methodologies [13,89–91]. However, in the present study, we could identify several ILs that showed transgressive segregation even under suboptimal and zero fertilizer application, especially for LC. The genotypes of the mapping populations DP-1 (LC-I and LC-II ≥24.10%, LC-III ≥80.83%, LC-IV ≥27.50%), DP-4 (LC-I and LC-II ≥23.80%, LC-III ≥76.10%, LC-IV ≥23.80%), and DP-7 (LC-I ≥60.01%, LC-II ≥34.52%, LC-III ≥80.01%, LC-IV ≥36.31%) have significantly exceeded the performance of the parents and checks. However, a total of 11 ILs, two ILs from DP-1 (*GSR-IR2-1-R11-L1-L2*, *GSR-IR2-1-R12-L1-R2*), four ILs (*GSR-IR2-4-L7-Y1-L2*, *GSR-IR2-4-R4-S1-L2*, *GSR-IR2-4-Y10-L1-Y2*, and *GSR-IR2-4-Y12-L1-Y2*) from DP-4, and five ILs (*GSR-IR2-7-L4-SU1-Y2*, *GSR-IR2-7-L8-SU2-R2*, *GSR-IR2-7-R13-SU1-L2*, *GSR-IR2-7-Y11-SU3-R2*, and *GSR-IR2-7-Y11-SU3-Y2*) from DP-7, have been commonly identified as promising ILs that show constitutively increased LC at DGI stages. These ILs, being highly responsive to fertilizer application, could be an imperative source of NuUE traits for future breeding programs and also an excellent source for the genetic dissection of tolerance of low inputs in rice. These potential ILs are highly useful for medium to low input marginal farmers who may not incur much of their cost in buying fertilizer for rice cultivation. Therefore, the area and production of rice would increase, which could further help in ensuring food security and farmers’ livelihood. This is one of the remarkable findings and applications of the present study and could be attributed to the contribution of the putative QTLs detected from the potential genetic backgrounds.

### Promising QTLs and comparison with previous QTLs related to deficiency of N, P, and K fertilizers

The rice Gramene database (http://archive.gramene.org), QTL Genome Viewer (http://qtaro.abr.affrc.go.jp), and previous reports from comprehensive literature surveys showed hundreds of QTLs associated with morphological, physiological, and biochemical traits that influence individual nutrient fertilizer deficiency in rice [10,41,52,92]. Among the 19 M-QTLs detected in the present study, 14 M-QTLs were detected in the same genomic region that was previously reported. These QTLs were significantly associated with more than 20 traits reported under N, P, and K deficiency tolerance, and were located on all chromosomes except chromosomes 7 and 8. The remaining five QTLs were found to be novel (Table 7). Among the 14 QTLs associated with FATs under DGI stages, nine QTLs were related to PH-I, LC-I, and LC-II, and four stages to TN on seven different chromosomes (2, 3, 4, 5, 6, 8, and 11) under deficiency of complete fertilizer (0 kg ha^‒1^). Six QTLs were related to PH-I, PH-III, PH-IV, TN-III, and LC-II on five chromosomes (1, 2, 3, 5, and 12) under suboptimal fertilizer dose (177.77 kg ha^-1^ N), and four QTLs were related to TN-I, TN-II, TN-III, and LC-II on four chromosomes (1, 9, 10, and 12) under the recommended dose of fertilizer, 100% (222.23 kg ha^‒1^ N). Some of these QTLs were co-localized with low N, P, and K tolerance on chromosomes 2, 4, 6, 10, 11, and 12. These QTL clusters might play an important role in NuUE in rice, and their related SNP markers could be useful for MAS in low input breeding programs.

**Table 7:** M-QTLs sharing similar genomic regions with previously reported QTLs.

S. no.	FD^a^ (% NPK)	QTL designation	QTL region (Mb)	Common region shared with earlier reported QTLs	
				QTLs	Reference
1	0%	*qLC-I_2*	29.07–28.88	*qRPH* ^¥^, *qRSDW*^¥^, and *qRKUP*^¥^	[68]
2	0%	*qTN-III_3*	27.66–29.5	*qDWR_3*^*#*^, *qNL_3*.*1*^*#*^,*qNL_3*.*2*^*#*^, *qaNUE_3*^*#*^, *qSP_3b*^*#*^, *qGD_3a*^*#*^, and *qFGp_3a*^*#*^	[58,93]
3	0%	*qTN-II_4*	31.28–32.42	qRRN_4*, OsPTR5*, and OsPTR6*	[98]
4	0%	*qPH-I_4*	15.43–21.82	*qSB_4*^*#*^ and qRRW_4*	[57,67]
5	0%	*qTN-IV_5*	17.8–18	*qDWR_5*^*#*^, *agNUE_5*^*#*^,*qDWS*^*#*^, *qDM*^*#*^, *qFW*^*#*^, *qTN*^*#*^, and *qaNUE*^*#*^	[93]
6	0%	*qTN-I_5*	17.80–17.86	*qDWR_5*^*#*^ and *qagNUE_5*^*#*^	[93]
7	0%	*qTN-II_6*	2.37–10.28	*qSP-6a*,*b*^*#*^, *qNCm6-12*^*#*^, *qNCD-6*^*#*^, qPUP*, *qRRDW_6*.1*, *qRSDW_6*.2*, *qRTDW_6*.3*, *qRPUC_6*.4*, *qRRL_6*.4*, and *qRWRSR_6*.6*	[53,58,64,67,94]
8	0%	*qLC-II_8*	8.21–8.92	Novel	-
9	0%	*qLC-II_11*	3.07–19.65	qRRDW_11*, *qPNP-11*.1*, qRWRSR*, and *qRRDW-11_1*^*#*^	[65,67,99]
10	80%	*qLC-II_1*	6.15–24.88	*qPN1*.7*, qSPF*	[67,98]
11	80%	*qPH-III_2*	30.45–30.56	Novel	-
12	80%	*qPH-IV_3*	0.24–1.24	Novel	-
13	80%	*qPH-I_5*	17.53–18.57	Novel	-
14	80%	*qTN-III_5*	17.47–17.80	Novel	-
15	80%	*qTN-III_12*	0.85–2.97	*qRTHK_12_1*^*#*^ and *qRDW_12*^*#*^	[99,100]
16	100%	*qLC-II_1*	29.88–33.6	*qRRDW_1_1*^*#*^, and *qSRL_1_2*^*#*^	[99,101]
17	100%	*qTN-I_9*	20.78–20.84	qDRW_9_5^*#*^	[100]
18	100%	*qTN-II_10*	4.70–14.56	*qNAA_10*^*#*^ and qPUP*	[53,102]
19	100%	*qPH-III_12*	5.91–6.59	Novel	-

^**a**^Fertilizer dose

^**b**^Percentage of phenotypic variation explained by each QTL

^**#**^QTLs for nitrogen deficiency

^*^QTLs for phosphorus deficiency

^**¥**^QTLs for potassium deficiency in rice.

By comparing with the previously reported QTLs, most of the QTLs were sharing common genetic regions at DGI stages in all populations. The QTLs were associated with other multiple QTLs, ranging from one to 11 under N, P, and K deficiency tolerance (Table 7). In the zero percentage of fertilizer condition, a total of three putative QTLs related to relative PH (cm) (*qRPH*), relative shoot dry weight (g/p) (*qRSDW*), and relative potassium uptake (mg/p) (*qRKUP*) shared the same genomic region with *qLC-I* on chromosome 2. Among the three QTLs, *qRPH* had the highest phenotypic variation of 14.70%, with an LOD score of 4.04, identified by using 123 DH lines under low-K stress conditions [68]. On chromosome 3, six QTLs related to N concentration in leaf sheaths plus stems (*qNS%_3*.*1*), N concentration in leaf blades (*qNL%_3*.*2*), dry weight of roots (*qDWR_3*.*3*), grain density per panicle (*qGD_3a*), number of filled grains per panicle (*qFGP_3a*), and number of spikelets per panicle (*qSP_3b*) shared the same genetic region with *qTN-III_3* at 29.07–28.88 Mb [58,93]. These QTLs were responsible for nitrogen use efficiency (NUE) and were also associated with yield-attributed traits under low nitrogen rates. Two genetic regions on chromosome 4 at 15.43–21.82 Mb and 31.28–32.42 Mb region of two QTLs (*qPH-I_4* and *qTH-II_4*) were shared with three QTLs related to shoot and root growth traits under low nitrogen and phosphorus tolerance and another two of them were related to phosphorus transporters [57,67]. By using the F_9-10_ generation of 169 recombinant inbred lines (RILs) derived from a cross between IR64 (*O*. *sativa* L. var. *indica*), and Azucena (*O*. *sativa* L. var. *japonica*), Thi et al. [93] identified 44 QTLs for 15 agronomic and NUE-related traits on all chromosomes, except chromosome 9. Among these traits, seven NUE-related traits such as agronomic NUE, number of tillers, total fresh matter, dry weight of roots, total dry matter, dry weight of sheaths plus stems, and dry weight of leaf blades were shared with the current study for two QTLs (*qTN-I_5* and *qTN-IV_5*) on chromosome 5 at 17.80–17.86 Mb region and 17.80–18 Mb region, respectively. However, the highest number of QTLs (11 QTLs) for low nitrogen and phosphorus tolerance were shared with *qTN-II_6* at the 2.37–10.28 Mb region on chromosome 6. Earlier, under low nitrogen conditions, Hu et al. [94] identified three QTLs for nitrogen content in shoots by using 116 DH populations, which have been developed through anther culture of F_1_ hybrids from *indica* rice variety Taichung Native 1 (TN1) and *japonica* rice variety ChunJiang 06 (CJ06). One of the main-effect QTLs, *qNCm6-12* (nitrogen content in plant shoots at mature stage), is significantly associated with the present QTL related to TN at stage II of fertilizer application. These QTLs mapped between RM527 and RM3 and explained PV of 9.73%. In addition, Feng et al. [58] identified 28 QTLs for yield-attributed traits on seven chromosomes under low N conditions. Two QTLs (*qPL-6a* and *qSP-6a)* for panicle length and number of spikelets per panicle explained PV of 15.58% and 6.40% from the analysis of 138 F_14_ RILs, which were derived from a super hybrid rice (Xieyou 9308) in China. Another QTL related to absorbed NUE (*qaNUE*) was also located in the same position [93]. The remaining seven QTLs were associated with low P tolerance QTLs. Of these, *qPUP_6* was associated with P uptake [95], four QTLs (*qRRDW*, *qRSDW*, *qRTDW*, and *qRPUC*) for root traits and P uptake in rice [64], and two QTLs (*qRRL*, and *qRWRSR*) for root growth and weight traits [67]. Interestingly, to date, there are no reported QTLs matched to/shared with the currently identified major QTL *qLC-II_8* (PVE of 22.85%) at the 8.21–8.92 Mb region on chromosome 8, which indicates that these QTLs are novel loci controlling for LC. However, Shimizu et al. [62], Wang et al. [96], and Tong et al. [97] reported 11 QTLs associated with LC on chromosomes 1, 2, 3, 4, 7, and 12 under low N and P conditions. As compared to the above studies, the current study identified four QTLs for LC on chromosomes 8 and 11 that are novel genetic regions under zero fertilizer conditions. The individual QTLs for LC had PVE ranging from 10.79% to 30.62%.The alleles from WTR-1 were in the direction of increasing the LC. These results indicate that these DGI stages of fertilizer application and cluster QTLs were significantly associated with low N, P, and K tolerances in rice.

Under suboptimal doses of fertilizer, a total of six QTLs (*qLC-II_1*, *qPH-III_2*, *qPH-IV_3*, *qPH-I_5*, *qTN-III_5*, and *qTN-III_12*) were associated with LC, TN, and PH located on five different chromosomes (1, 2, 3, 5, and 12), explaining an average PV of 19.09%. The smallest genetic region of *qPH-III_2* (0.11 Mb) was located on chromosome 2, and *qPH-IV_3* (1 Mb) was located on chromosome 3. Two QTLs, *qPH-I_5* (1.04 Mb) and *qTN-III_5* (0.33 Mb), were located on chromosome 5, and those are not shared with any reported QTLs. Therefore, these QTLs were considered as novel loci for DGI stages of fertilizer application. The remaining two QTLs, *qLC-II_1* on chromosome 1 and *qTN-III_12* on chromosome 12, at 6.15–24.88 Mb region and 0.85–2.97 Mb region, were shared. A total of three QTLs were related to low N and two QTLs to low P tolerance traits. Three QTLs (*qTNCS_1b*, *qSY_1b*, and *qGD_1a*) under low N on chromosome 1 shared the same genetic region with LC-II_1. The first two QTLs for total nitrogen content of shoot and straw yield were noticed by Cho et al. [54] by using 166 F_8_ lines derived from a cross between a Korean *tongil* type of rice, variety Dasanbyeo, and a Chinese *japonica* variety, TR22183. Similarly, Feng et al. [58] identified three QTLs for grain density (GD) per panicle by using 138 F_14_ RILs, under low N. One of the QTLs, *qGD_1a* (6.04% PVE) located on chromosome 1, was shared with *qLC-II_1*. Two other QTLs (*qRTDW_1* and *qRSDW_1*) were shared with *qLC-II_1* on chromosome 1. These two QTLs were associated with low P tolerance. Lastly, chromosome 12 at the 0.85–2.97 Mb region showed the presence of two QTLs (*qRTHK_12_1* and *qRDW_12*) for root thickness [100] and root dry weight [99] by using RILs under low N conditions. On the other hand, for the recommended dose of fertilizer, four QTLs (*qLC-II_1*, *qTN-I_9*, *qTN-II_10* and *qPH-III_12*) located on four different chromosomes (1, 9, 10, and 12) had considerable association with earlier reports of several QTLs related to low P and N tolerance in rice.

For yield-attributed and NUE traits related to QTLs under low N conditions, Lian et al. [99] and Dai et al. [102] reported a total of 15 main-effect QTLs for root and shoot weight and 13 QTLs distributed on all 12 chromosomes. Of these, two QTLs, for relative root weight, *qRRW_1a* [99], and seminal root length, *SRL_1*.*2* [101], on chromosome 1 shared a common genetic region with *qLC-II_1*. Similarly, a single QTL, *qDRW-9*.*5* [100], on chromosome 9 was shared with *qTN-I_9* under low N conditions. The genetic region of 4.70–14.56 Mb associated with *qTN-II_10* is shared with two QTLs: for nitrogen accumulation amount, *qNAA_10* [102], and phosphorus uptake, *qPUP_10* [53]. Lastly, chromosome 12 at the 5.91–6.59 Mb region is associated with *qPH-III_12* (PVE of 23.06%), which is not shared with any previous reports of QTLs and is considered to be a novel locus. In summary, among all the shared genetic regions, QTLs detected on chromosomes 1, 4, 5, 10 and 11 confer N and P deficiency tolerance, those on chromosomes 3 and 4 confer N deficiency tolerance, those on chromosome 4 confer P deficiency tolerance, and those on chromosome 2 confer K deficiency tolerance in rice. The clusters of QTLs detected under different doses of fertilizer at DGI stages and novel QTLs are valuable genetic resources to identify useful genes and introgression in elite lines to improve rice in NuUE.

### Putative candidate genes and functions

The development of highly nutrient-responsive genotypes for low nutrient application is imperative during this era when the area and production of rice are under threat. MAS is one of the main strategies for accelerating the process of developing lines with higher NuUE. Effective MAS using major-effect QTLs mainly depends on the highest co-segregation of the markers and trait. Understanding of the molecular mechanisms and gene structure of the loci detected increases the efficiency of MAS. Hence, in the present study, we explored the candidate genes within the major-effect loci interval region (≤1 Mb) and their predicted molecular and physiological functions. A total of eight putative QTLs detected on five different chromosomes (2, 5, 8, 9, and 12) were explored for the candidate gene analysis survey (Table 7). A total of 120 genes were identified in the promising QTL interval regions. Each QTL has a variable number of underlying associated genes ranging from 3 genes (58.2 kb) on chromosome 3 to 30 genes (711.90 kb) on chromosome 8. *In silico* analysis revealed that 78.33% of the genes are functionally annotated while the remaining genes are expressed and hypothetical proteins. The list of all possible candidate loci and their functions is detailed in S2 Table and Fig 5. These genes were functionally related to numerous physiological and molecular functions such as photosynthetic rate, synthesis of chlorophyll precursors, phosphate transporters (peptide transporter), zinc transporters, growth stimulators of auxin-responsive genes, abscisic acid (ABA) signaling pathways, and activation of various transcription factors, all of which were found within the promising M-QTL interval regions.

A QTL (*qPH-II_2*) located at 28.88–29.07 Mb on chromosome 2 associated with PH at stage II of fertilizer application was neighboring the candidate gene *Os02g46140*, which encodes for F-box protein domain involved in several biological functions, is related to phytohormone signaling pathways, and regulates various developmental processes as a part of the abiotic stress response mechanism [103]. Another candidate gene, *Os02g46090*, is associated with calcium signaling pathways. Several families of calcium sensors have been reported and they have played major roles in nitrogen metabolism and abiotic and biotic stress response mechanisms [104]. Recently, researchers identified a novel mutant of the calcium-dependent protein-kinase-encoding gene, *esl4*, which is significantly expressed in roots, shoots, and enzyme activity of several genes related to nitrogen metabolism [105]. Interestingly, two candidate genes, a tonoplast-localized low-affinity nitrate transporter, *OsNPF7*.*2* [106], and a transcriptional regulator, GROWTH-REGULATOR FACTOR 4 (*OsGRF4*), along with a growth inhibitor of DELLA proteins [107], are located in the same genetic region on chromosome 2. The hotspot region of the root-specific transporter plays a vital role in intracellular allocation of nitrate in roots, especially in sclerenchyma, cortex, and stele, and ultimately leads to influences on plant growth. The interaction of GRF4 and DELLA proteins is involved in physiological activities to regulate multiple genes for carbon and nitrogen metabolism, plays a significant role in the homeostatic coordination of nitrogen metabolism, and also increases shoot biomass and PH [107,108]. Overexpression and mutant analysis of *OsNPF7*.*2* showed that the significant expression of *OsNPF7*.*2* caused an increase in TN by regulating the cytokinin and strigolactone pathway [109]. Another QTL, *qLC-I_2* at the 30.45–30.56 Mb region, was found to be located on chromosome 2, adjacent to another candidate gene, *OsGS1;1* (cytosolic glutamine synthatase), which regulates nitrogen metabolic pathways and also influences plant growth and development [110,111]. The GS1 proteins have a major role in generating glutamine, which is the primary form of remobilized nitrogen during natural senescence in leaves for long-distance transport [112]. In addition to that, *qLC-I_2* is adjacent to the *diacylglycerolacetyl transferase* (*OsDGAT*) (*Os02g48350*) gene and interacts with *OsTCP19* in regulating seedling establishment by modulating stress signaling molecules and abscisic acid pathways in various abiotic stresses [113].

Three QTLs, *qPH-I*, *qTN-III*, and *qTN-IV* (17.47–18.00 Mb), were found to be located on chromosome 5 adjacent to *Os05g30970*, and they encode for copine-like protein. It is mainly involved in the nutritional role of glutamate for aiding in seedling establishment under nitrogen deficiency [114]. In the same position are three other possible putative candidate loci: *Os05g30870* (*OsRLCK185*), for diverse roles in plant growth and development and stress responses [115]; and *Os05g30240*, encoding for pentatricopeptide-repeat protein for upregulation in salt stress conditions [116]. Hence, to overcome nutrient deficiency, balanced mineral nutrients are essential for optimal plant growth and development. Each of these genes/loci played a critical role in various physiological and molecular responses such as cytokinins negatively regulating Pi (phosphate) starvation and also regulating metabolic changes under low N [117,118], and phytohormones such as auxins and seconday metabolites that are involved in the maintenance of homeostasis, root hair development, and signaling pathways under low NPK tolerance mechansim [119,120]. Similarly, phytohormones, ABA, jasmonates (JA), and their associated biosynthetic genes at vegetative and reproductive stages regulate various signaling pathways, leading to adaptation to nutrient deficiency through the root architecture, and maintaining N, P, and K homeostasis [121–123]. The *in silico* expression analysis was performed for the candidate genes, *Os02g46140*, *Os02g46090*, and *Os02g48350* on chromosome 2 and *Os05g30970*, *Os05g30870*, and *Os05g30240* on chromosome 5 located within M-QTL regions using RiceXpro [124]. The expression analysis indicated wide differential expression pattern of these candidate genes (S2 Fig). The high level of expression was observed in ovary development for the two candidate genes, *Os02g46090* and *Os05g30240*, whereas for the other genes high level of expression was noticed in spikelet hull (lemma and palea) during the early stages of seed development and higher expression during roots and panicles development. Hence, these candidate genes within the M-QTL interval region can be considered as the promising putative candidate genes. However, further validation and developing functional markers are necessary for their effective application in breeding programs.

## Conclusions

Genetic dissection of low N, P, and K tolerance at DGI stages of fertilizer application is an important target area for modern breeding programs in rice. Hence, in the present study, we identified the genomic regions that confer low N, P, and K tolerance for favorable agronomic traits at different stages of fertilizer application and selected ILs possessing the highest tolerance of low NPK using three different MPs derived from three donors (HAN, CH448, and Z413). ANOVA and descriptive statistics indicated the existence of significant genetic variation among the FATs and the predominance of non-Mendelian inheritance. To identify the genetic factors that influence these quantitatively inherited traits, QTL analysis was carried out by using the precisely estimated phenotypic values and high-quality SNPs derived from the tGBS genotyping platform. A total of 19 M-QTLs on different chromosomes were detected. Among these M-QTLs, eight QTLs were considered as putative QTLs, with the smallest locus size (≤1 Mb) contributing the highest toward trait expression. Among the putative QTLs, *qTN-I_92*, *qTN-III_5*, *qLC-I_2*, and *qLC-II_8* detected under 100%, 80%, and 0% NPK were found to be responsible for transgressive segregation of the ILs for the trait. Notably, *qLC-I_2*, *qTN-IV_5*, and *qLC-II_8* detected at zero fertilizer application showed higher performance for LC under 0% of NPK fertilizer. These QTLs not only help in building a tolerance of low N, P, and K nutrient simultaneously but also improve genotypes to make them highly responsive to lower nutrient application. This is one of the remarkable achievements of the current study, which helps low-input and marginal farmers to cultivate rice without incurring high fertilizer cost and to eventually ensure food security and sustainable agriculture. On the other hand, *in silico* functional annotation of candidate genes within the putative QTLs indicated that two and five candidate genes found to confer tolerance of low N, P, and K and related to several physiological and metabolic pathways were also found to be involved in abiotic stress tolerance. However, additional investigation is needed for further confirmation to examine the potential physiological and molecular mechanisms. These studies can help in understanding the underlying complex genetic interactions involved in nutrient use efficiency and the identification of more efficient breeding materials containing these genetic factors. Furthermore, the detected genomic regions related to stage-specific tolerance of low fertilizer doses and promising ILs can be useful for MAS and future breeding programs for low-input conditions.

## Supporting information

S1 Table M-QTLs detected for all FAT at DGI stages under different doses of fertilizer. (XLSX)

S2 Table Possible putative candidate genes in QTL regions. (XLSX)

S1 Fig Overall mean performances of all ILs under three different doses of fertilizer at DGI stages. (TIFF)

S2 Fig Graphical representation of six putative candidate genes expression profiles, derived from the spatiotemporal expression profiling at vegetative and reproductive stages as available in RiceXpro database. (TIFF)
