# Supplementary figures and images for: Genetic dissection of developmental responses of agro-morphological traits under different doses of nutrient fertilizers using high-density SNP markers
Source: PLoS One. 2019 Jul 23;14(7):e0220066. doi: 10.1371/journal.pone.0220066 (PMC6650078; doi:10.1371/journal.pone.0220066)

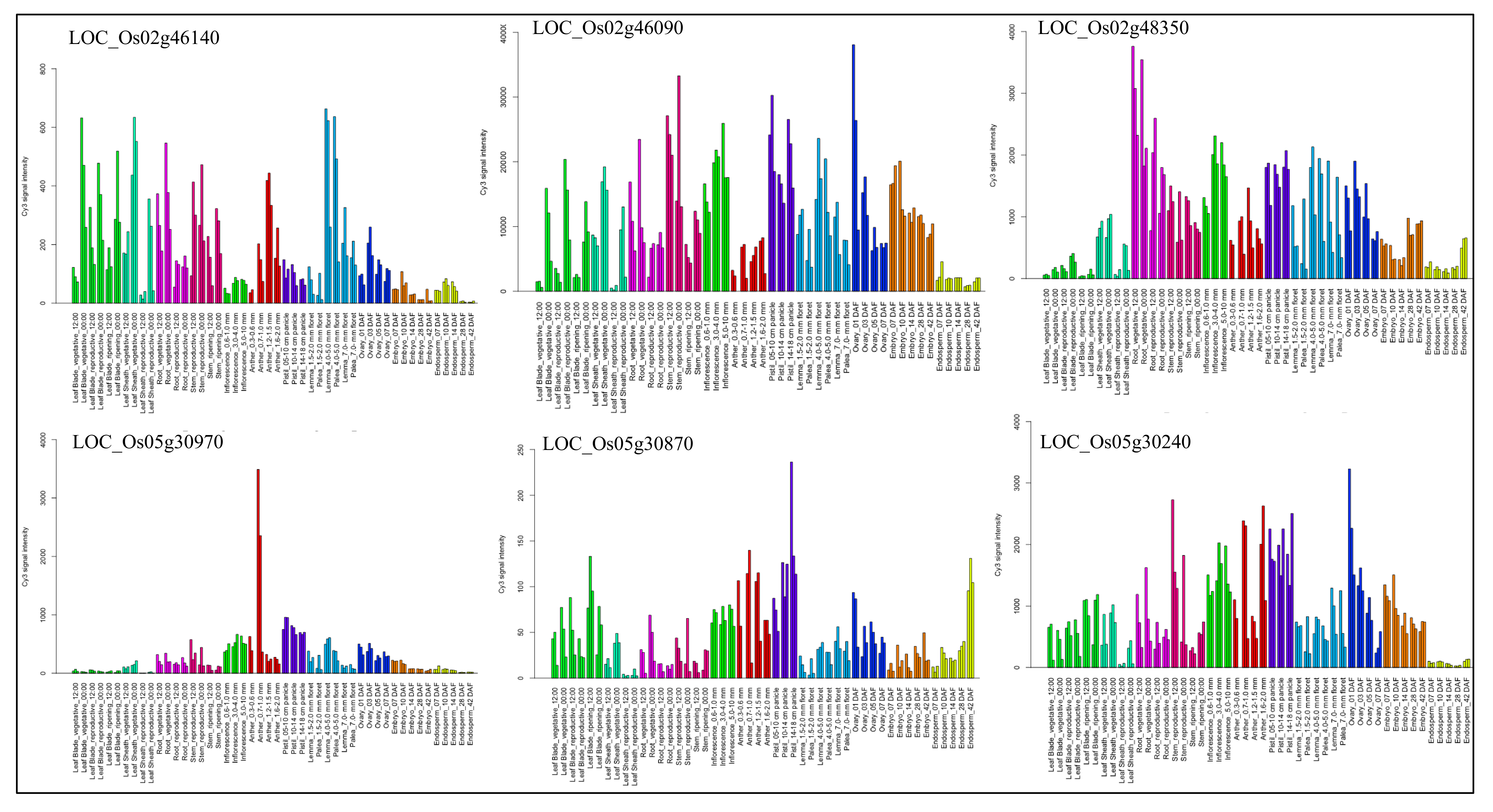

Supplement: S2 Fig — (TIFF) [file pone.0220066.s004.tiff]
